# Judging Strangers’ Trustworthiness is Associated with Theory of Mind Skills

**DOI:** 10.3389/fpsyt.2015.00052

**Published:** 2015-04-20

**Authors:** Marie Prevost, Mathieu Brodeur, Kristine H. Onishi, Martin Lepage, Ian Gold

**Affiliations:** ^1^Department of Philosophy, McGill University, Montreal, QC, Canada; ^2^Department of Psychiatry, Douglas Mental Health University Institute, McGill University, Montreal, QC, Canada; ^3^Department of Psychology, Montreal, QC, Canada

**Keywords:** paranoia, schizophrenia, trust, delusions, theory of mind, reading the mind in the eyes test

## Abstract

Trusting people requires evaluating them to assess their trustworthiness. Evaluating a stranger’s intentions is likely to be one method of assessing trustworthiness. The present study tested the hypothesis that judgments of trustworthiness are associated with mind reading skills, also called theory of mind (ToM). We tested a group of healthy participants and a group of patients with paranoid schizophrenia. Both groups made ToM judgments and judged the trustworthiness of strangers. Participants were also assessed for their disposition to trust as well as levels of paranoid belief. As anticipated, healthy participants had a normal ToM scores and patients with paranoid schizophrenia had poor ToM scores. In paranoid patients, better ability to read others’ minds was associated with judging others as more trustworthy, while the reverse was found in the healthy participants (better mind reading was associated with judging others as less trustworthy), suggesting a non-linear relationship between trust in others and being able to read their intentions.

## Introduction

The decision to trust someone could be equivalent to making the judgment that the person is trustworthy (1). In turn, judging that someone is trustworthy depends in part on assessing that person’s intentions from the available information (2). If judgments of trustworthiness depend on intention reading, then the ability to read others’ minds [an instance of the capacity often called “theory of mind” or ToM; (3)] should be associated with judgments of trustworthiness – a relationship that has, to our knowledge, not yet been investigated empirically. Thus, the first objective of the present study was to explore the relationship between being able to read others’ intentions and judging other as trustworthy or not.

Trust is essential for social (4) and economic well-being (5). Evaluations of trustworthiness are often constructed from three characteristics (2, 6, 7): (1) the person’s competency, which relates to a sense of the person’s control over a situation and an ability to deal with complex situations; (2) the person’s integrity, which relates to a moral dimension and expecting the person to act as she says she will; and (3) the person’s intentions, which relates to that person’s benevolence or good intentions. However, even when we meet people for the first time (thus before having much information about them), we can quickly evaluate their trustworthiness (8, 9). For example, people are judged as trustworthy when their faces were also rated as attractive, intelligent, or not aggressive (10). Recent research has investigated the relationship between the perception of basic emotional facial expressions and trustworthiness (9, 11–13), but little is known about the relationship between judgments of trustworthiness and the ability to read more subtle mental states.

In the current study, we asked whether perception and inference of subtle mental states similarly correlate with ratings of trustworthiness. To assess the ability to read mental states, we used the reading the mind in the eyes test [RMET (14)]. The RMET involves looking at pictures of strangers’ eyes and choosing one of four words that best describes what the person in the picture is feeling or thinking, and is taken to be a measure of our ability to perceive and infer someone’s emotional or intentional states (14). Specifically, we tested whether RMET performance is associated with judgments of trustworthiness.

Although no studies have directly examined the link between reading intentions and judgments of trustworthiness, previous findings provide some suggestive information. Teenagers who were good at taking another person’s perspective were more trusting and punished untrustworthy partners to a greater degree than teenagers who were poor perspective-takers (15). Similarly, students with high empathy skills, a form of ToM (16, 17), were more trusting of others than those with low empathy skills (18). We might thus expect that people who are good at reading intentions would also be more trusting. Consistent with a link between mind reading and trust, fake smiles were associated with lower trustworthiness evaluations than authentic smiles (19), suggesting that perception and inference of intentions (at least falsely conveyed ones) are indeed related to trustworthiness judgments. Together, the previous studies suggest that better perception of strangers’ intentions (ToM) is likely to be associated with ratings of trustworthiness.

In the normal population, ToM skills are generally high although there is some variability (14). So, to test a wide range of ToM skills, we tested two groups of participants: healthy individuals who we anticipated would score normally on ToM tests, and patients with paranoid schizophrenia. Paranoia is characterized by persecutory delusions, defined as “belief[s] that one is going to be harmed, harassed, and so forth by an individual, organization, or other group” [DSM-V (20)]. Impaired ToM has been repeatedly observed in patients with schizophrenia (21–23), and more specifically, in patients with paranoid schizophrenia (24–27). We thus anticipated that these patients would perform poorly on the RMET (28, 29), which we used as a measure of ToM, allowing to test the hypothesis that impaired ability to read others’ intentions would be associated with less trust in others as well. If so, these findings could help define new therapeutic avenue for schizophrenia and paranoia; symptoms might be reduced by working on improving the cognitive and behavioral processes of trust in these patients.

It has been assumed that paranoid schizophrenia patients have issues with trust given that they (by definition) perceive others as threats, and given that recent indirect empirical links between mistrust and schizophrenia have been found [e.g., Ref. (30, 31)]. For example, during a financial investment task patients with schizophrenia displayed lower trust, investing less money overall (32, 33). This decreased trust behavior compared to controls tended to be correlated with positive psychotic symptoms, supporting the idea of an association between paranoia and trust (32). In addition to lower levels of trust overall, the patients (unlike healthy participants) were unable to modulate their trust behavior based on evidence. Thus, healthy participants, but not patients, adjusted their behavior based on both specific information provided about a partner’s trustworthiness and based on direct evidence of that person’s trustworthiness in a multi-round investment game.

Thus, the primary objective of the current study was to examine the link between being able to read the intentions of others and trusting others. Specifically, we predicted that (1) lower ToM skills would be found in the paranoid schizophrenia group compared to the healthy participants, (2) lower ToM skills would be associated with lower levels of trust (across the two groups), and (3) levels of trust might be mediated differently in the two groups, since healthy participants but not patients would be better at detecting and using evidence of trustworthiness. A second objective of the present study was to verify the link between paranoid schizophrenia and trusting others.

## Materials and Methods

### Participants

Patients with clinical paranoia (P; *n* = 13; 7 male) were recruited from outpatient units at the Douglas Mental Health University Institute in Montreal, QC, Canada. They met the criteria for non-affective psychotic disorder (schizophrenia and other non-affective psychosis) and had been treated for a minimum of 4 years. Their current primary diagnosis was paranoid schizophrenia or their score on the persecutory delusion subscale of the scale for assessment of positive symptoms [SAPS; (34)] was three or more. Exclusion criteria were a lifetime history of medical or neurological conditions that affect cognition; a family history of hereditary neurological disorders; a diagnosis of substance dependence; the presence of depression; and being pregnant. Positive and negative symptoms were assessed by a certified psychologist using the SAPS (34) and the scale for assessment of negative symptoms [SANS; (35)]. As all patients were medically treated, we report chlorpromazine equivalent following Woods (36) in Table 1.

**Table 1:** **For each group, means (SDs) for demographic, clinical, and experimental measures are reported**.

	P (*n* = 13)	H (*n* = 14)	H minus P [95% confidence interval]
**Demographic information**
Age	33.6 (9.1)	34.8 (7.5)	1.1 [−5.6; 7.9]
Education	10.4 (3.1)	12.9 (1.7)	**2.5 [0.4; 4.6]**
Intellectual quotient	96.2 (15.1)	109.1 (12.8)	**12.9 [1.7; 24.1]**
**Clinical information**
Age of onset	22.2 (6.0)	–	–
Medication	713.6 (526.1)	–	–
Scale for assessment of positive symptoms	26.1 (15.2)	–	–
Scale for assessment of negative symptoms	25.3 (13.9)	–	–
**Experimental results**
Paranoia scale	39.1 (10.1)	23.8 (3.8)	**−15.3 [−21.6; −8.9]**
Trust scale	9.7 (2.3)	10.7 (1.2)	1.0 [−0.4; 2.4]
Reading the mind in the eyes test	20.3 (5.3)	26.0 (3.6)	**5.7 [2.0; 9.3]**
Trustworthiness	5.4 (1.4)	6.3 (1.1)	*0.9 [−0.1; 1.9]*

*In addition, we show mean differences between paranoid schizophrenia patients (P) and healthy participants (H). Medication is in chlorpromazine equivalents*.

*Significant (*p* < 0.05) differences are in bold*.

*Trends are in italics (*p* < 0.1)*.

Healthy participants (H; *n* = 14; 9 male) were recruited through internet advertising. Exclusion criteria were the same as for patient groups with exclusion also for those with a history of any Axis-I disorders and with a parent with a diagnosis of schizophrenia.

All participants received (1) the structured clinical diagnosis for axis-I disorders – DSM-IV to either confirm the diagnosis (Patient Edition) or exclude any other mental illness (SCID-I) and (2) the Wechsler adult intelligence scale [WAIS; (37)] to assess intellectual quotient (IQ).

### Tasks and scales

The *Paranoia scale* (38) was developed to assess the tendency to believe that others want to harm you. It is composed of 20 items (e.g., “Someone has been trying to influence my mind”) which the participant rates on a scale from 1 (not at all applicable to me) to 4 (extremely applicable to me), for a total score ranging from 20 to 80. The scale has excellent internal consistency (Cronbach’s α = 0.84), and stability over time (6 months) is high (*r* = 0.70). The validity of the scale is very good, as its scores correlate significantly with other paranoia scales in healthy participants (39) and with clinical ratings of paranoia in patients (40).

The *trust in strangers scale* (41) is a scale that measures trust in strangers versus trust in known others or in institutions. It consists of four items (e.g., “In general, you can trust people”) rated by the participant on a scale from 1 (disagree strongly) to 4 (agree strongly), for a total score ranging from 4 to 16. It has good internal consistency (Cronbach’s α = 0.66), and its stability over time is moderate (over a 6-week interval; *r* = 0.48). Validity is good, with moderate correlations with self-reported measures of trust from the European Social survey (*r* = 0.47), the Neuroticism-Extraversion-Openness Personality Inventory-Revised (NEO-PI-R; *r* = 0.55), and the General Social Survey (*r* = 0.53).

The *reading the mind in the eyes test* [RMET; (14)] evaluates ToM. Participants are shown black and white pictures of the eye region of strangers together with four words describing different mental states and are asked to choose the word that best captures the mental state (feelings, thoughts) of the person pictured. The test consists of 36 items, with 1 given for choosing the target word and 0 for choosing any of the 3 foil words, for a total score ranging from 0 to 36.

We developed a novel *judgment of trustworthiness task* using the stimuli from the RMET so that ratings of the strangers’ trustworthiness could be directly connected to RMET scores. For each picture in the RMET, participants were asked to judge the trustworthiness of the person on a scale from 1 (not trustworthy) to 10 (very trustworthy), by pressing on 1 of the 10 keys assigned for each integral value from 1 to 10. The final score was the mean judgment of trustworthiness, and thus ranged from 1 to 10.

### Procedure

On visit 1, the SCID-I, SAPS, SANS, and WAIS were administered by a psychologist during a clinical evaluation as part of a larger study. On visit 2, participants completed the Paranoia Scale, the Trust in Strangers Scale, the RMET, and the Judgment of Trustworthiness task. All participants gave written informed consent. The research was approved by the Douglas Institute’s Research Ethics Committee.

### Analyses

For each participant, we calculated the total score for each measure. We report means for each group and the mean differences between the two groups with 95% confidence intervals (CI), and compare the two groups of participants using independent samples *t*-tests. Correlation coefficients and *p*-values are reported for bivariate correlations between the different measures within each group.

For the link between reading intentions and judgments of trustworthiness, we had three main predictions.

**Figure 1:**
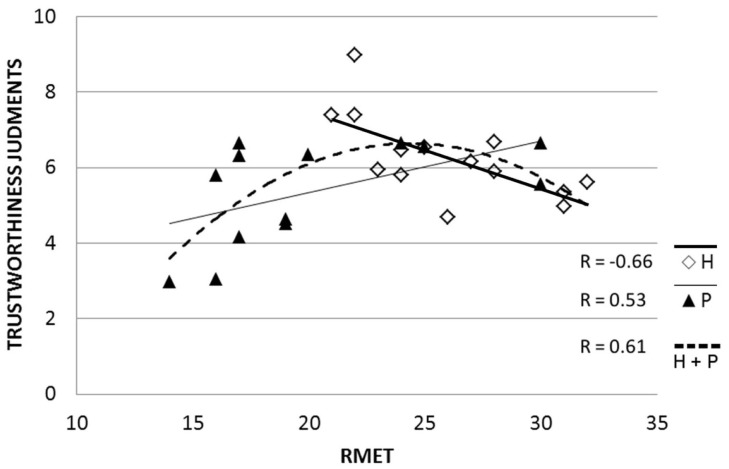
**Correlations between RMET scores and Judgments of Trustworthiness in patients with paranoid schizophrenia (P) and healthy participants (H)**. Linear regressions are displayed for both groups. A second order polynomial model collapsing across patients and healthy participants (dashed line) is also shown.

(1) To verify that lower ToM skills would be found in paranoid schizophrenia participants than in healthy participants, we compared RMET scores between the groups using an independent samples *t*-test. To further explore this difference, we separated the 36 items into positive, negative, and neutral mental states, based on Harkness et al. (42): 12 mental states are categorized as negative, 8 mental states are categorized as positive, and 16 mental states are categorized as neutral. In these cases, we reported RMET performance in percentage instead of the mean total scores since the number of items included in each valence group of items was not identical.
(2) To test the relationship between RMET and judgments of trustworthiness, we combined all the participants, based on the assumption that there is a continuum of behaviors and mental processes from schizophrenic individuals through healthy individuals (43–46). For the combined group, a second order polynomial model was used given the dispersion of the data (Figure 1).
(3) To explore whether levels of trust might be differentially mediated by RMET scores within the two groups we fit a linear regression between RMET and judgment of trustworthiness scores for each group separately (Figure 1). This question was further examined by exploring the correlations between RMET scores and judgments of trustworthiness for positive, negative, and neutral items.

To address the second objective and thus to verify the link between paranoid schizophrenia and trust in others, we compared the mean scores at the Trust in Strangers scale and for judgments of trustworthiness between groups using independent samples *t*-tests.

## Results

Paranoid patients were similar in age to the healthy participants but were less educated and had lower IQs. Mean scores for each group and mean differences between patients and healthy participants are shown in Table 1. Controlling for IQ and education did not change the differences observed (Paranoia scale mean adjusted difference = −7.05, *p* = 0.001; RMET mean adjusted difference = 2.31, *p* = 0.039).

Healthy participants performed significantly better on the RMET than patients with paranoid schizophrenia, confirming our prediction that lower ToM skills would be found in patients than in healthy people. Exploring further this difference, we observed that for the neutral pictures of the RMET, paranoid patients were less accurate (51%) than healthy participants (75%; CI −95% = [14–34%], *p* = 0.001), but for positive (patients = 67%; healthy participants = 78%) and negative (patients = 56%; healthy participants = 65%) items there was no difference between groups.

To test the relationship between RMET and judgments of trustworthiness scores, we treated patients and healthy participants as a single group (*n* = 27), and a polynomial model of second order (see Figure 1) was used rather than a linear model given the dispersion of the data. Following this model, judging others are trustworthy was associated with average RMET scores.

In fact, separate analyses for each group (see Figure 1) revealed that RMET scores and trustworthiness judgments tended to be correlated in paranoid patients (*r* = 0.53, *p* = 0.061) and were correlated in healthy controls (*r* = −0.66, *p* = 0.010) but in opposite directions (Fisher’s *z* = 2.52, *p* = 0.011). Thus, for paranoid patients, the better they were at reading others’ minds, the more trustworthy they tended to judge others. For healthy controls, in contrast, better mind reading was associated with judging others as less trustworthy. RMET scores did not significantly correlate with the paranoia score in either group. In addition, groups were different in how they used the specific cues available in the pictures for reading others’ intentions. For the negative pictures, healthy participants showed a significant negative relationship between their RMET and judgments of trustworthiness scores (*r* = −0.62, *p* = 0.019) whereas paranoid patients did not (*r* = 0.39, *p* = 0.19). All other correlations were not significant.

To test whether paranoia and trust were related, we investigated differences in, as well as correlations between, paranoia and trust scores. As expected (see Table 1), healthy participants had lower scores on the paranoia scale than the paranoid patients and healthy participants tended to judge others as more trustworthy than the paranoid patients did. However, the groups did not differ on Trust scale scores. In addition, scores on Paranoia and Trust were not correlated for either group and SAPS scores in patients did not correlate with Trust score or Judgment of trustworthiness.

## Discussion

The first objective of this study was to explore whether good ToM skills are associated with trusting others by exploring whether reading others intentions and judgments of trustworthiness are linked. Trusting others requires taking risks (47), and these risks might be minimized if one has the ability to understand others’ intentions. Our results partially support this hypothesis. First, as expected healthy participants were better mind readers than paranoid patients, confirming previous reports (24–26). Second, participants who were very poor mind readers (mostly patients) judged others as untrustworthy (Figure 1). Third, ToM skills and judgments of trustworthiness were not related in the same way in each group: in healthy participant RMET performance was negatively associated with judgments of trustworthiness, whereas in paranoid patients, this relationship tended to be reversed (a non-significant positive correlation between RMET scores and trustworthiness).

As expected, we found that the link between reading others’ intentions and judging their trustworthiness was mediated differently in our two groups. Healthy participants who were good mind readers tended to judge strangers as neither trustworthy nor untrustworthy, staying near the midpoint of our 10 point scale. In these participants, the influence of detecting positive intentions might not weight more than the influence of detecting negative intentions. When looking at the negative mental states of the RMET, a negative correlation between judgments of trustworthiness and RMET scores was observed, meaning the participants who were skilled at detecting strangers’ negative intentions judged them as untrustworthy. The reverse (judging strangers as more trustworthy when participants were good at detecting their positive intentions) was not significant, confirming that idea. One limitation here is that healthy and paranoid participants did not have the same range of scores on the RMET. Thus, we do not know how healthy participants who are very poor mind readers would have evaluated strangers’ trustworthiness. On the contrary, we found no evidence that the patients’ judgments of trustworthiness were mediated by the valence of the pictures, which is consistent with the idea that low mind-reading skills are associated with an inability to use all the available information [e.g., Ref. (32)]. For example, in Fett et al. (32), schizophrenia patients did not modulate their trust behavior toward a partner based on explicit information about the partner’s trustworthiness or based on the partner’s behavior. Our results are consistent with the idea that patients did not perceive and infer intentions from facial signal as well as healthy participants (who had higher RMET scores), and might not use these signals from the faces to guide their judgments of trustworthiness. This conclusion is based on null results with a small sample size and on a correlational analysis and thus must be explored further in the future.

However, at present, our results are consistent with the hypothesis [e.g., Ref. (2)] that ToM skills are associated with trustworthiness evaluation (at least for negative mental states). The current results echo previous findings showing that people will judge strangers as trustworthy if they express positive emotions such as happiness and as untrustworthy if they express negative emotions such as anger (9, 10). We showed here that trustworthiness can be associated with the perception of mental states and not only to the perception of basic emotional states. Just as reading the emotions of others may enhance communication and cooperation, reading their intentions is also critical (48) and this cooperation will be more likely to occur if trustworthiness signals are accurately perceived (49).

Our second objective was to confirm that paranoid patients are less trusting than healthy participants (32, 33). Paranoid patients and healthy participants did judge strangers’ trustworthiness differently, with paranoid patients tending to provide lower judgments, somewhat supporting this idea. However, there was no difference between patients and healthy participants on the Trust scale scores. There is a possibility that no differences are observed on this scale due to a lack of awareness from patients regarding their own predisposition to distrust others. Indeed, the Trust scale is a self-reported measure asking whether we trust people in general. Patients might be aware that they mistrust one particular person when asked about that (as in the Trustworthiness Judgment task), but when asked about people in general, they might feel trusting and not be aware that their general tendency is toward mistrust. This is reminiscent of a recent study, which showed that schizophrenia patients were not in tune with their empathic skills, contrary to healthy participants (50). Other studies are needed to better explore this question.

To conclude, healthy participants were better at reading others’ intentions than were paranoid schizophrenia patients. The relationship between mind reading and trusting others was different in the two groups. In healthy participants, good mind reading was associated with judging strangers cautiously as neither trustworthy nor untrustworthy and this seems to be due to the detection of negative intentions or mental states to guide judgment of trustworthiness. On the contrary, in paranoid patients, the relationship between mind reading and trustworthiness was only a trend, suggesting that patients might not use their ToM to guide their trustworthiness evaluation. Finally, general levels of paranoia levels did not predict average scores for trusting others, suggesting that the association between trust and paranoia should be further examined before assuming that there is necessarily a link between paranoia and trust. The current results add to the limited empirical evidence testing the hypothesis that to trust a stranger, we need to have a sense of that stranger’s intentions (2, 6).

## Author Contributions

All authors contributed to the design of the study, the interpretation of the results, and the multiple revisions of the present manuscript. MP tested the participants and analyzed the data. All authors approved the final version, and agreed to be accountable for all aspects of the present work.

## Conflict of Interest Statement

The authors declare that the research was conducted without commercial or financial relationships that could be construed as a potential conflict of interest.
